# Wireless, battery-free, fully implantable multimodal and multisite pacemakers for applications in small animal models

**DOI:** 10.1038/s41467-019-13637-w

**Published:** 2019-12-17

**Authors:** Philipp Gutruf, Rose T. Yin, K. Benjamin Lee, Jokubas Ausra, Jaclyn A. Brennan, Yun Qiao, Zhaoqian Xie, Roberto Peralta, Olivia Talarico, Alejandro Murillo, Sheena W. Chen, John P. Leshock, Chad R. Haney, Emily A. Waters, Changxing Zhang, Haiwen Luan, Yonggang Huang, Gregory Trachiotis, Igor R. Efimov, John A. Rogers

**Affiliations:** 1 Department of Biomedical Engineering, University of Arizona, Tucson, AZ 85721 USA; 2 Center for Bio-Integrated Electronics at the Simpson Querrey Institute for BioNanotechnology and the Department of Materials Science and Engineering, Northwestern University, Evanston, IL USA; 3 Department of Biomedical Engineering, The George Washington University, Washington, DC USA; 4 Department of Surgery, The George Washington University, Washington, DC USA; 5 Department of Engineering Mechanics, Dalian University of Technology, Dalian, 116024 China; 6 Department of Civil and Environmental Engineering, Mechanical Engineering, and Materials Science and Engineering, Northwestern University, Evanston, IL 60208 USA; 7 Department of Aerospace and Mechanical Engineering, University of Arizona, Tucson, AZ 85721 USA; 8 Department of Biomedical Engineering, McCormick School of Engineering and Applied Science, Northwestern University, Evanston, IL USA; 9 Center for Advanced Molecular Imaging, Radiology, and Biomedical Engineering, Northwestern University, Evanston, IL USA; 10 Center for Advanced Molecular Imaging, Northwestern University, Evanston, IL USA; 11 AML, Department of Engineering Mechanics, Interdisciplinary Research Center for Flexible Electronics Technology, Tsinghua University, Beijing, 100084 China; 12 DC Veterans Affairs Medical Center, The George Washington University, Washington, DC USA; 13 Departments of Materials Science and Engineering, Biomedical Engineering, Neurological Surgery, Chemistry, Mechanical Engineering, Electrical Engineering and Computer Science, Simpson Querrey Institute and Feinberg Medical School, Center for Bio-Integrated Electronics, Northwestern University, Evanston, IL 60208 USA

**Keywords:** Cardiovascular diseases, Biomedical engineering

## Abstract

Small animals support a wide range of pathological phenotypes and genotypes as versatile, affordable models for pathogenesis of cardiovascular diseases and for exploration of strategies in electrotherapy, gene therapy, and optogenetics. Pacing tools in such contexts are currently limited to tethered embodiments that constrain animal behaviors and experimental designs. Here, we introduce a highly miniaturized wireless energy-harvesting and digital communication electronics for thin, miniaturized pacing platforms weighing 110 mg with capabilities for subdermal implantation and tolerance to over 200,000 multiaxial cycles of strain without degradation in electrical or optical performance. Multimodal and multisite pacing in ex vivo and in vivo studies over many days demonstrate chronic stability and excellent biocompatibility. Optogenetic stimulation of cardiac cycles with in-animal control and induction of heart failure through chronic pacing serve as examples of modes of operation relevant to fundamental and applied cardiovascular research and biomedical technology.

## Abstract (web summary)

Pacing tools that support small animals and can serve as models for pathogenesis of cardiovascular diseases are currently not available. Here, the authors report a miniaturized wireless battery-free implantable multimodal and multisite pacemaker that provides unlimited stimulation to test subjects.

## Introduction

Animal models play critical roles in investigations of pathogenesis of cardiovascular disease and in studies of multifactorial contributions to cardiac health, including genetic and environmental factors, which take years to fully manifest in humans. Research on these mechanisms can be accelerated by utilizing small animals, native or genetically engineered. Here, natural or induced rapid heart rates lead to comparatively quick pathophysiological processes resulting in cardiovascular diseases, such as atrial fibrillation and pacing-induced heart failure^1–3^. Outcomes of experiments in these contexts, including chronic investigations with constrained parameters that cannot be controlled in studies of affected human populations^4^, can provide insights into fundamental mechanisms.

Pacemakers, originally developed as clinical treatments for abnormal heart rhythms, have also been harnessed to reveal functional cardiac pathology and to explore the adverse effects of ectopic pacing on cardiac electrophysiology. For example, continuous, rapid pacing in large animal models can induce pathological remodeling of cardiac electrophysiology and tissue architecture, thereby significantly contributing to our understanding of pathogenesis of atrial fibrillation^5,6^ and heart failure^7,8^. Recently developed techniques based on optogenetics can effectively pace and defibrillate^9–12^ by utilizing light to optically stimulate genetically targeted, light-activated proteins (for example, channelrhodopsin 2 (ChR2)) to depolarize cardiac cells^13^. Optogenetic cardiac and autonomic nerve stimulation, in contrast to electrical pacing, allows for functional dissection by selective stimulation of specific neuron or cardiomyocyte subpopulations^14^.

Small animal models offer optimal versatility in the exploration of cardiovascular diseases across a span of phenotypes and genotypes. Significant advantages of small animal models, especially mice, follow from the ability to engineer genes that encode various cardiovascular proteins with critical roles in normal and pathological physiology. Current methods to provide cardiac stimuli to small animal models almost exclusively feature a physical tether for electrical power supply and user control, thereby limiting investigations to anesthetized in vivo studies of immobilized animals. Devices that feature optogenetic interfaces to the heart are in their infancy, as most are derived from those employed for optogenetic techniques in the brain^9^. Here, delivery of illumination occurs through optical fiber technologies borrowed from the telecommunication industry, with limited suitability for conscious, freely moving animal models or long-term in vivo studies. The high-modulus rigid nature of traditional optical fibers can limit insights in in vivo observations and frustrate chronic studies^15^, particularly in rapidly moving, soft tissues systems such as those of the heart. Use of freely roaming, behaving, and interacting animal models in naturalistic environments demand the development of electrical and optical pacing tools that are highly miniaturized, powered wirelessly, and configured in mechanically compliant forms to deliver pacing stimuli in small animal models for chronic studies in the field, without limits in operational lifetimes^16^. Absent from current literature are multimodal and multisite stimulation devices of this type that enable electrical and/or optical stimuli, in forms analogous to those for the brain, the peripheral nerves, the spinal cord, and the bladder^17–19^. Cardiac devices are of particular interest, because they offer the opportunity to study chronic effects of multisite/multimodal cardiac pacing and neuromodulation, resulting in pathogenesis of heart diseases.

Here, we present a class of device that supports optical and electrical multisite stimulation in engineering designs that address these requirements, with formats that allow full subdermal implantation in small animal models such as rats and mice. The battery-free operation and control schemes rely on wireless interfaces that exploit magnetic resonant coupling. This feature omits the weight and bulk associated with battery packs, along with the needs to replace and/or recharge, thereby allowing for indefinite operation with minimal influence on natural animal behaviors. The tether-free nature of such devices also allows for paradigms with fully conscious, freely moving subjects, in isolation or in social groups.

## Results

### Implantable, wireless, battery-free multimodal multisite pacemaker

**Fig. 1:**
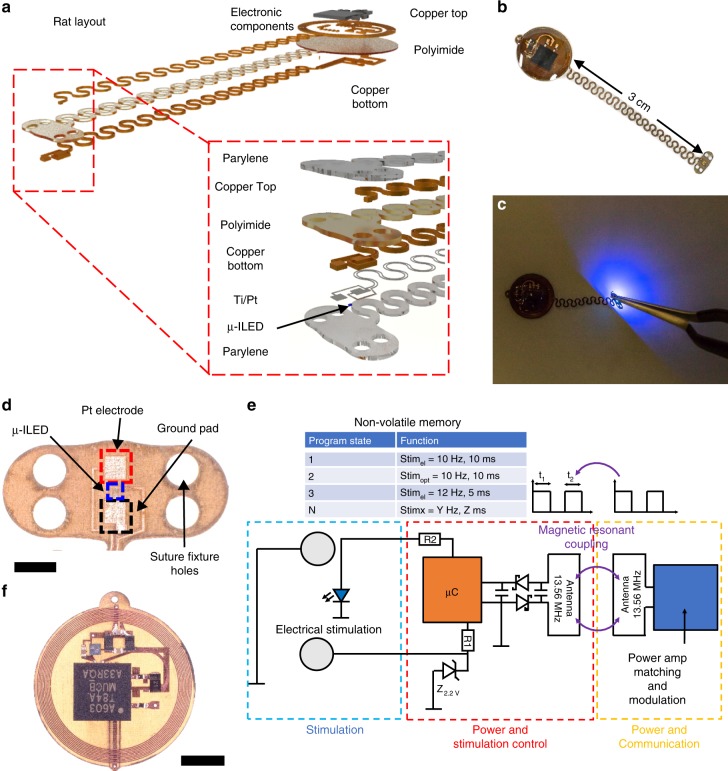
Wireless, battery-free, fully implantable pacemakers with electrical and optical stimulation capabilities. **a** Rendered images of the layered designs of devices configured for rats and mice. **b** Photographic images of the devices. **c** Photographic image of device activated for optogenetic stimulation capability. **d** Optical micrograph of the stimulation electrode (scale bar 1 mm). **e** Schematic illustration of the operating principles. **f** Microscopic image of the receiver and control unit of the device (scale bar 3 mm).

Figure 1 summarizes the key mechanical and electrical features of the device, the latter of which exploits custom communication protocols and power regulation techniques described in Gutruf et al.^19^. Digital communication with the implant allows independent activation of electrical and optical stimulation from multiple sites. The electronics for energy harvesting and stimulation control reside on a circular platform that couples to a monolithically defined stretchable interconnect that terminates in the stimulating optoelectronic interface, as shown in the exploded view schematic illustration in Fig. 1a and the photograph in Fig. 1b. The layered construction relies on a polyimide (PI) flex circuit substrate and a layout that both align with established manufacturing processes, thereby facilitating opportunities for rapid dissemination to the scientific community. The stimulation electrode consists of platinum-coated copper. A conformal coating of parylene C serves as an encapsulation layer. In related platforms, such coatings support lifetimes of up to 1.5 years^20^. Detailed information on the materials and fabrication can be found in the Methods section. The lateral dimensions are sufficiently small to allow for full subdermal implantation in rats. A schematic illustration of the device layout with dimensions and detailed information on electrical components is in Supplementary Fig. 1. Figure 1c shows a photograph of the microscale inorganic light-emitting diode (µ-ILED) during irradiance that exceeds 10 mW/mm^2^, sufficient to stimulate ChR2-expressing hearts used in this study. During operation of the µ-ILED, the temperatures of the adjacent tissues remain well below the thresholds for damage and for activation^19^. Figure 1d shows an optical micrograph of a representative optrode, as defined by co-located electrical and optical stimulators, on a platform that enables fixation to the target tissue via suture holes optimized for quick placement on a rapidly beating rat heart in vivo (300–500 b.p.m.).

Figure 1e illustrates the electrical working principles. Optical micrographs of the harvester and stimulation control are in Fig. 1f. The secondary antenna on the implant harvests radio frequency (RF) power from a primary antenna that encircles the experimental arena via magnetic resonant coupling. Power is harvested via a single wave rectifier that includes a matching capacitor for tuning the system to an operational frequency of 13.56 MHz. The harvested power, conditioned by a linear regulator, provides a stable supply to the microcontroller (µC) that controls electrical and optical stimulus. The former is limited by a Zener diode that caps voltages exceeding 2.2 V to avoid damage to the epicardium. The current to both electrical and optical stimulation elements is limited via resistor R1 and R2. The resulting geometry is highly miniaturized and lightweight (~110 mg).

### Mechanical and electrical characterization

**Fig. 2:**
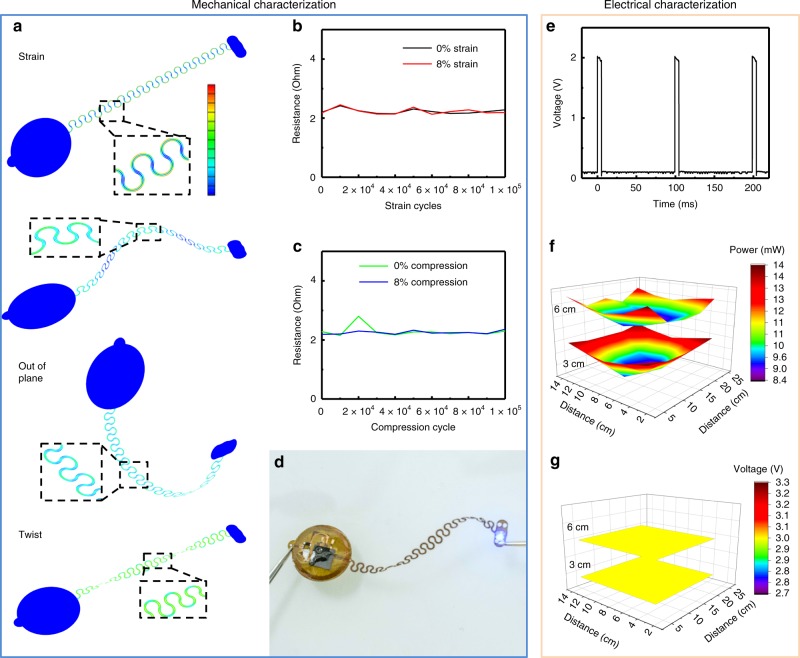
Mechanical and electrical characterization results. **a** Finite-element simulation results for **b** 100,000 cycles of uniaxial tensile strain between 0 and 8%, with resistance measurements. **c** 100,000 cycles of uniaxial buckling from compressive strains between 0 and 8% stain. **d** Photographic image of a twisted device with activated optogenetic stimulator (scale bar 10 mm). **e** Time-resolved voltage of pacing electrodes. **f** Spatially resolved wireless resonant power in a test cage. **g** Spatially resolved regulated voltage with device load.

Figure 2 characterizes the electrical and optical properties of the system. The design supports a mechanically robust, stretchable interconnect between the harvesting and control electronics and the optrode. The interface must remain stable over repeated strain cycles due to the oscillatory motion of the epicardium relative to the thorax, which corresponds to a cyclic strain of ~8% in rodents^21^. Finite-element analysis (FEA) informs designs that facilitate stretchable mechanics well within the elastic limit and below the yield strain of the copper, to ensure extensive cyclic stability. Figure 2a shows deformations associated with stretching, buckling, out of plane bending, and twisting of the interconnect structure. For all cases, the strains remain below 0.3% in the copper, its limit for plastic deformation. Figure 2b shows the resistance of the traces for the interconnects to the stimulation electrodes, shorted at the electrodes and the inputs to the power and control module. The resistances in the strained and relaxed states show stable values for 100,000 cycles to strains of 8%, similar to those expected in the myocardial motion of a rat heart^21^. Subjecting the same sample to an additional 100,000 cycles of 8% compression results in multiple deformations in an upward buckling mode, as shown in the FEA simulations. The resistance measurements displayed in Fig. 2c indicate stable behavior across all scenarios examined. The ultimate failure mode of this same sample occurs upon stretching to a strain of ~150%, via mechanical fracture, as shown in Supplementary Fig. 2a. Cycling above the elastic limits, which are not expected during normal operation in the animal, results in a slow degradation and ultimate failure. As demonstrated in Supplementary Fig. 2b, cycling to strains of 12% results in a gradual increase of resistance due mainly to microcracking in the copper^22^. Even in this case, the conductivity supports function for over 100,000 cycles of strain. Figure 2d shows operation of the device under extreme twisting, which is unlikely to occur in vitro. Experimental and FEA results suggest that deformations even to this extent remain well within the elastic limits.

Electrical characterization shows high timing accuracy during wireless and battery-free operation (Fig. 2e), facilitated by a wireless power supply that exceeds the power consumption of the device (typically ~6 mW peak during optical stimulation, depending on output setting) at any location within the experimental arena. Figure 2f shows a two-dimensional (2D) spatial map of power available at 3 and 6 cm from the cage floor. Figure 2g additionally shows a stable system voltage regulated by the linear dropout regulator, for any location within the cage, similar to that obtained with related devices and similar antenna systems^19^.

**Fig. 3:**
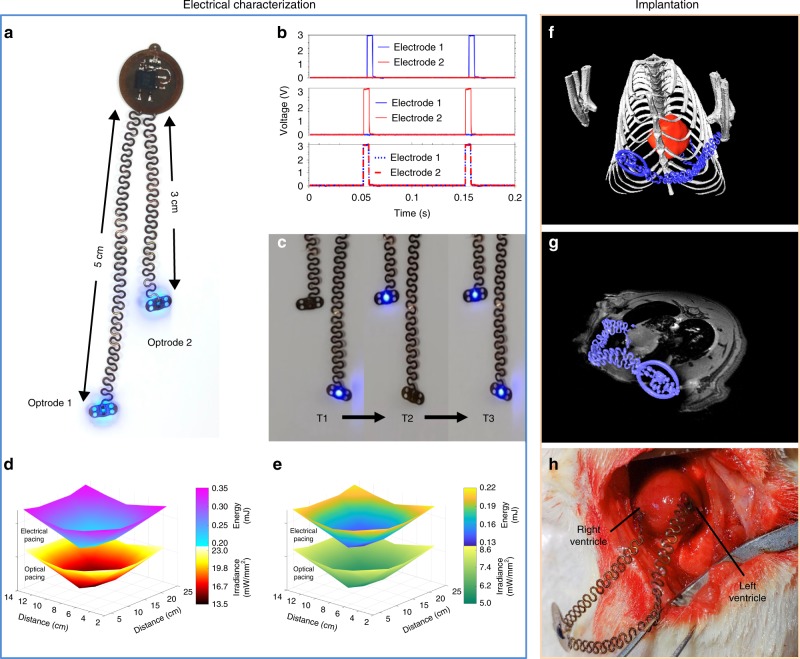
Multisite pacing. **a** Photographic image of a bilateral optogenetic stimulator. **b** Time-resolved voltage of pacing electrodes in a unilateral pacing mode for electrode 1 and 2, and in a bilateral pacing mode. **c** Independently addressable bilateral optogenetic stimulation at intervals T1, left optrode activation, T2 right optrode activation, and T3 simultaneous activation. **d** Spatially resolved energy and irradiance distribution of individual optrodes for independent bilateral electrical and optical pacing in a test cage at a height of 3 cm from the floor. **e** Spatially resolved energy and irradiance distribution for individual optrodes for combined bilateral electrical and optical pacing in a test cage at a height of 3 cm from the floor. **f** 3D rendering of an implanted device in a rat derived from combined MRI and CT images. **g** Combined 3D CT and 2D MRI slice of a bilateral pacemaker implanted in a rat, indicating epicardial positioning of optrodes. **h** In vivo *s*urgical implantation of the device on a rat heart.

Although heart failure can be induced in patients and animal models via single-site pacemakers, due to unphysiological excitation patterns, biventricular pacing is used clinically to achieve more ventricular synchrony via multisite pacing. Results in Fig. 3 demonstrate multisite pacing, a modality critically important to induce synchronous multimodal pacing for a variety of heart failure models, which is clinically used for resynchronization therapy. In addition, such multimodal/multisite pacing can be used for cardiac and neural stimulation with multimodal optrodes capable of electrical and optical stimulation, to study cardiac neuromodulation. Figure 3a shows a photograph of a biventricular pacemaker with active optogenetic stimulators. The dimensions facilitate full subdermal implantation for stimulating the anterior epicardial surface of the left and right ventricles of a rat model independently. Figure 3b illustrates capabilities with representative stimulation patterns. Here an activation pattern with a frequency of 10 Hz and time duration of 5 ms appears with either electrode 1 or 2 and in conjunction. Accurate timing enabled by the µC also allows for asynchronous pacing with frequency and duty cycles chosen with microsecond precision.Optical stimulation is displayed in Fig. 3c, where independently addressable bilateral optogenetic stimulation for the left optrode activation (T1), right optrode activation (T2), and simultaneous activation (T3) can be achieved with selectable duty cycles and frequencies.

Device output features control of stimulation intensity via duty cycle modulation. The maximum stimulation capabilities depend on the power that can be harvested wirelessly. The results in Fig. 3d illustrate the relationship between stimulation output, stimulation mode, and spatial location. Here, the available energy and irradiance for bilateral stimulation of either electrodes or µ-ILEDs are shown at a vertical location of 3 cm from cage floor. Measurements correspond to power availability for electrical and optical pulses with durations of 5 ms. Simultaneous activation of both stimuli can occur for specific pacing patterns. Figure 3e indicates the available energy and irradiance for this combined electrical and optical pacing with 5 ms pulses at a cage height of 3 cm. Supplementary Fig. 3 shows stable operation of regulated device output voltage in experimental arenas for rats.

A three-dimensional rendering of the implanted device in a rat from magnetic resonance imaging (MRI) and computed tomography (CT) scans is in Fig. 3f. Figure 3g is a combined CT and 2D MRI slice of the bilateral pacemaker implanted in a rat in the transverse plane. Figure 3h shows an open chest view of the biventricular surgical implantation of the bilateral device on the rat heart in vivo. Each optrode is sutured to the anterior surface of the epicardium of the left and right ventricle, respectively.

### Implantation procedure and biocompatibility

**Fig. 4:**
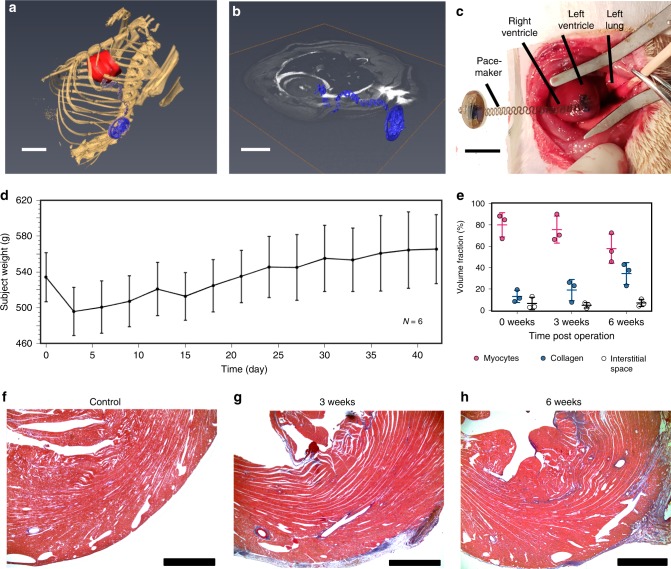
Device implantation and biocompatibility. **a** 3D segmentation of anatomical positioning of device (blue) with respect to the rat heart (red) (scale bar 1 cm). **b** Anatomical positioning of the device (blue) visualized in the transverse plane (scale bar 1 cm). **c** The device is sutured to the anterior epicardial surface of the left ventricle of the rat (scale bar 1 cm). **d** Animal subjects were postoperatively weighed for 3 (*n* = 3) and 6 weeks (*n* = 3). Error bars indicate mean ± SD. **e** The transmural anterior left ventricle was stained with Masson’s trichrome in adult rats. No significant differences in fibrosis were found between any time points (*p* < 0.05). Error bars indicate mean ± SD. **f** Representative images of the transmural anterior left ventricle without pacemaker implantations (*n* = 3) (scale bar 1 mm) and at (**g**) 3 weeks (*n* = 3) (scale bar 1 mm) and (**h**) 6 weeks after pacemaker implantation (*n* = 3) (scale bar 1 mm).

In the example of Fig. 4a–c, the electrodes attach to the anterior epicardial surface of the left ventricle and the device receiver resides in the subcutaneous space. For 13 implanted devices, all but 1 animal survived the procedure (mortality rate, 7.7%). The cause of death was myocardial infarction and fatal arrhythmia following an injury to the left anterior descending coronary artery during the implantation. The weight of the animals decreased by 7.2 ± 1.3% in the first 3 days following the surgery. The weight stabilized thereafter and all animals regained their original weight by the postoperative day 6, with weight gain appropriately in subsequent days (Fig. 4d). No other major postoperative complications occurred in the surviving animals. Assessment of device biocompatibility by Masson’s trichrome staining showed no significant differences in myocardial volume fraction indicative of no major myocardial damage secondary to the implanted device. The level of fibrosis in the tissue area surrounding the site of attachment did not significantly increase (Fig. 4e). Representative stained histological sections images at 0, 3, and 6 weeks post implantation appear in Fig. 4f–h. Explantation of the device 6 weeks reveals complete healing of the skin at the incision site, with the device and coil fully intact (Supplementary Fig. 4). The device was incorporated well into the native tissue with no observable signs of apparent rejection responses in the surrounding tissues. Similar level of biocompatibility was reported previously from conventional epicardial pacemakers in pigs^23^. The weight of the animals in our study initially declined, as expected after a major surgery, but normalized to pre-implantation values and continued to increase with aging over the course of several weeks after surgery, indicating that the animal subjects healed well in response to the foreign device.

### Ex vivo electrical pacing characterization

**Fig. 5:**
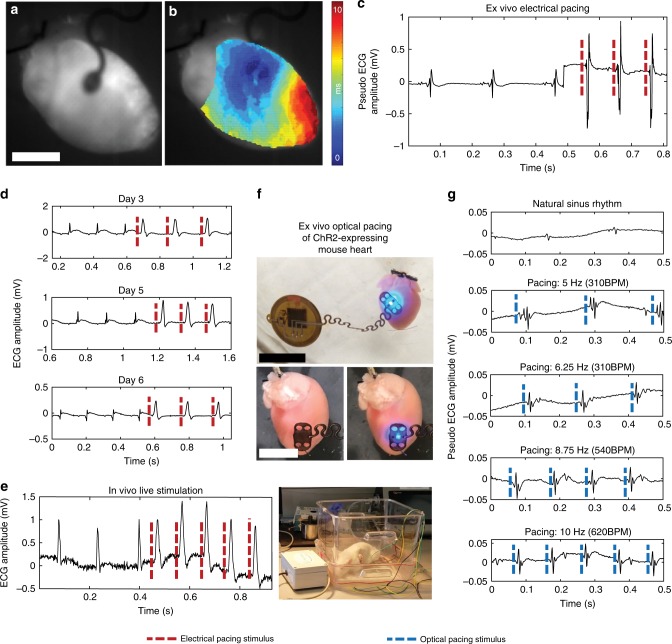
Electrical and optogenetic pacing capabilities. **a** Mouse hearts were electrically stimulated by the device ex vivo (scale bar 0.5 cm). **b** The time course of pacing activation was tracked using the membrane potential to show anisotropic conduction by optical mapping. **c** Far-field ECG pacing demonstrates capture of the heart during ex vivo pacing. **d** Chronic in vivo pacing of rat hearts was achieved with implanted pacemakers for up to 6 days. **e** Rat hearts were captured (right) while the animal was freely moving. **f** Ex vivo ChR2-expressing mouse hearts were optically paced at the anterior epicardial surface of the left ventricle (top) (scale bar 1 mm) in the off (bottom left) and on (bottom right) configuration (scale bar 0.5 mm). **g** Ex vivo ChR2-expressing mouse hearts were able to be captured at 280, 310, 540, and 620 b.p.m.

Functionality of electrical and optical pacing was verified in isolated mouse hearts expressing opsin ChR2 using optical mapping and far-field electrocardiography (ECG) recordings (Fig. 5). Contact of the activated pacemaker with the heart showed a visual increase in the frequency of mechanical contraction (Supplementary Movie 1). Activation maps of ex vivo electrical pacing show clear anisotropic activation of the membrane potential originating from the middle of the right ventricle where the device lead was placed (Supplementary Movie 2). The membrane potential activation clearly propagates throughout the ventricular myocardium (Fig. 5a, b). Additional information is provided in Supplementary Fig. 9. Electrical capture was simultaneously verified with ECG recordings. A clear increase in heart rate and QRS complex amplitude was observed with the onset of pacing to demonstrate that the device drives the heart rhythm (Fig. 5c).

### Chronic pacing capability

All animals with implanted pacemakers underwent daily pacing trials while awake or under light sedation (Fig. 5e and Supplementary Movie 3). Successful ventricular rapid pacing appears in the ECG tracings, as indicated by a narrow QRS that converts to a wide QRS with shortened *R*–*R* intervals. Use of a device with programmable pacing voltage via primary transmission coil RF output power modulation allowed for further studies. On day 0 and 1, a threshold power to the primary coil of 6–7 W enabled rapid ventricular pacing. This threshold increased by 0.5 to 1 W daily, as was observed in clinical pacing prior to advent of steroid eluting leads^24^. The trial was halted if the threshold increased above 10 W. The successful capture, e.g., the ability of successful external pacing of the heart, was observed up to postoperative day 6 (Fig. 5d). At day 7, the pacemaker continued to deliver electrical stimulus, verified by explanation of the device with subsequent electrical functionality tests, but failed to drive the heart to rapid ventricular pacing. Upon explantation, a normal point of contact of the pacing electrode to the epicardial surface of the heart was visually confirmed, for all devices. Examination revealed no signs of device migration, but some post-surgical adhesions were observed. In vitro testing following explantation indicated normal function, without degradation.

### Ex vivo optical pacing characterization

For ex vivo optogenetic pacing, a wireless device configured for blue light stimulation was placed on the surface of the ventricular myocardium in ChR2-expressing mice. Targeted µ-ILED stimulation involves illumination onto a portion of the excitable myocardium (Fig. 5f), the area of which is sufficient to induce an electrical response as verified by far-field ECG (Supplementary Movie 4). The device was programmed to deliver illumination at 5, 6.25, 8.75, and 10 Hz, and the heart rate was found to increase in correspondence with increasing illumination frequency (Fig. 5g). FEA reveals a minimal change in temperature arising from operation of the µ-ILED at the interface with the epicardium (Supplementary Fig. 5). The model only investigates diffusion and does not account for perfusion of the tissue, therefore representing a worst-case scenario. Temperature increases in vivo are likely to be significantly lower. Maximum temperature increases of 0.09 °C occur for frequency and duty cycles that elicit stimulation response (Supplementary Movies 5 and 6). This temperature increase is well within the 2 °C threshold of cardiomyocyte cell death, apoptosis, or necrosis^25^. The optrodes also produce significantly less thermal load in comparison with recent wired optogenetic activation via a high powered LED in the thoracic cavity where 0.2 °C of temperature increase was found to induce no physiological changes^26^, suggesting that the thermal emission by the optrode does not damage or modulate function other than in targeted cell groups.

Devices explanted after 6 weeks showed no evidence of damage on both macroscopic examination and histologic analysis, consistent with ex vivo mechanical stress testing and finite element modeling. Nevertheless, optical pacing was limited to 6 days post implantation due to gradual increases in the threshold power caused by the inflammatory response, as was routinely observed in the early days of clinical pacing^24^. The use of steroid eluting pacing leads, as studies of conventional devices^27^, might represent a solution to the limitation.

## Discussion

The classes of multimodal and chronically implantable pacemakers introduced here offer wireless and battery-free capabilities well suited for use in small animal models such as rats. The devices enable chronic pacing of freely moving subjects, without mechanical or functional degradation, in designs that are compatible with MRI and CT imaging. The ultralight, flexible, and monolithic construction offers excellent biocompatibility in designs that align well with manufacturing capabilities in flexible printed circuit board technologies, with the potential for broad dissemination.

Digital control over optical and electrical pacing parameters supports versatile capabilities in the study of behaviors in a given animal, over time. Long-term rapid electrical pacing has been shown to induce heart failure^28^. In addition, heart failure can be induced in rodents with surgical methods^29^ and genetic mutations^30^. Failure can also be induced by rapid pacing in large animals such as pigs^8,31,32^, dogs^7,33,34^, and sheep^35,36^, but genetic modifications are extremely difficult and costly. Rodents are a much more versatile animal model for transgenic studies. With only one exception (Laughner et al.^16^), previous electrical pacemakers were not sufficiently small to be fully implanted. The devices reported here overcome such limitations to provide a small, anatomically matched geometry that extends capabilities to targeted stimulation with high spatial and temporal precision via multimodal optrodes. Specifically, in addition to electrical stimulus, ChR2-expressing mice can be optically paced or neuromodulated with the same device, over a range of frequencies. In this way, long-term effects of optogenetic pacing can be studied, where the optical interface may mitigate complications associated with electrode/tissue contacts that are prone to gradual increases in impedance and inflammation responses that can sometimes lead to failures in chronic applications. In addition to pacing, optical defibrillation of ChR2-expressing hearts by LED stimulation can also be achieved by illuminating a larger part of the myocardium^10,26,37^ and is a subject of future investigation.

There are potential translational applications for this type of device. In current technology, the pacemaker battery makes up the majority of the device size and weight. This often results in a largely visible device that sits on the upper chest that causes significant amount of dissatisfaction and even reoperation for many patients around the world^38,39^. Even recently approved leadless pacing systems, which lack a traditional pacemaker box, still require battery power and periodic replacement^40^. Having multiple battery-free pacemakers that are wirelessly powered can significantly improve the quality of resynchronization therapy, as multiple wireless pacemakers can be programmed externally to synchronize cardiac excitation and contraction, with the potential to increase cardiac output and reduce adverse effects of traditional single-site right ventricular pacing.

## Methods

### Device fabrication

Pyralux AP8535R served as a substrate for the circuit, the serpentine interconnect and the optrode. The top and bottom copper layers (17.5 µm thick) and via holes were structured via direct laser ablation (LPKF U4). Through-hole plating was performed via pulsed direct current electroplating of copper (LPKF Contac U4), to define the electrical connections between the top and bottom layers. Electron beam evaporation of 200 nm platinum defines the electrode surface. Components with commercial packaging were attached via reflow soldering with low-temperature solder (IndiumCorp). The µ-ILEDs were mounted with a pick-and-place tool (Finetech Fineplacer pico ma) using defined force and temperature (180 °C) with an anisotropic conductive paste. The devices were encapsulated with coatings of parylene formed by chemical vapor deposition (CVD) (14 µm). Electrodes were masked with laser-defined Kapton during CVD process and removed manually to expose the electrodes. Commercially packaged components such as the µC and operational amplifiers were further encapsulated with PDMS (DOW corning Sylgard 184) to minimize any foreign body response. Fabrication of the biventricular device was similar to that of the single optrode pacemaker.

### Electronic components

Small outline, packaged off-the-shelf components served as the basis for the circuits. A low power µC (ATtiny 84 (Atmel)) with wide operational voltage capability provided current to the LED and voltage for the electrical stimulation, which was limited by a 2.2 V Zener diode. The µC firmware was programmed prior to device assembly. The µC settings for the biventricular device allowed for individual control of each optrode. A low-dropout (150 mV), low-quiescent current (18 µA) linear regulator with fixed internal output voltage (2.8 V) managed power to the implants (ON semiconductor NCP161). Passive components with 0201 and 0402 package size helped minimize the footprint.

### Mechanical testing

Mechanical testing was performed on a custom build stretching stage based on a screw-driven linear stage (Aerotech ATS100) with LabView programming interface.

### MRI and CT imaging

MRI was performed on a 9.4T Bruker Biospec MRI system with a 30 cm bore, a 12 cm gradient insert, and an Autopac automated sample positioning system (Bruker Biospin Inc., Billerica, MA). An actively decoupled four-channel, phased array receive-only radiofrequency coil designed for rat brain (Bruker Biospin, Inc., Billerica, MA) was mounted on the bed. This assembly was centered inside a 72 mm quadrature volume coil in transmit-only mode (Bruker Biospin, Inc., Billerica, MA). Rats were imaged using an accelerated spin echo sequence (Rapid Acquisition with Relaxation Enhancement, RARE) oriented in axial, sagittal, and coronal directions. The following parameters were used: repetition time (TR)/echo time (TE) = 2000 ms/40 ms, RARE factor 8, acquisition matrix (MTX) = 256 × 256, field of view (FOV) 2 × 2 cm, 11–17 slices of 0.75–1 mm thick (as needed for full brain coverage), and 2 signal averages. Fat saturation was disabled, as this was found to slightly reduce image artifacts from the implanted devices. Acquisition time was ~2 min per scan. The reconstructed data were visualized in Amira 6.4 (FEI, Houston, TX). MRI and microCT images were manually registered as the image artifacts caused by the device precluded automatic image registration. A non-local means filter was applied to the CT data in Amira to reduce image artifacts.

### Wireless operation of pacemaker devices

A commercial RF system (Neurolux, Inc.) was used to wirelessly deliver power to either electrical or optical pacemaking devices for whole heart stimulation. The system included the following: (1) a laptop with custom NeuroLux software to control and command the data center, (2) a Power Distribution Control box to supply wireless power and communication to the devices with interactive TTL inputs, (3) an antenna tuner box to maximize power transfer and match the impedance of the source and the antenna, and (4) an enclosed cage with customizable loop antenna designs for operation of the devices throughout a variety of settings (e.g., in vivo and ex vivo conditions) (Supplementary Fig. 6).

### Animals

All animal procedures were completed in agreement with The George Washington University institutional guidelines and in compliance with suggestions from the panel of Euthanasia of the American Veterinary Medical Association and the National Institutes of Health Guide for the Care and Use of Laboratory Animals. Transgenic mice intended for use in optogenetic experiments were crossbred to express ChR2 in cardiac myocardial cells using Cre-Lox recombination. One parent (Jackson Labs stock #011038) was a hemizygous mouse with Cre recombinase expression driven by the cardiac-specific alpha myosin-heavy chain promoter, whereas the other parent (Jackson Labs stock #024109) was a homozygous flox-ChR2-YFP mouse, which has enhanced yellow fluorescent protein (EYFP)-tagged *ChR2* that will express in the presence of Cre. Together, they create αMyHC-Cre-ChR2 mice which express ChR2 in the heart only. Genotyping of tail snips (Transnetyx) confirmed the genotype of properly expressing offspring (Cre+;Fl/Fl) to be used in experiments.

### Optical mapping of acute ex vivo electrical pacing

Optical mapping was performed on ex vivo isolated mouse hearts. Briefly, mice were deeply anesthetized with a mixture of 5% isoflurane/95% O_2_ until unconscious. Following cessation of pain reflexes, mice were injected with heparin (250 U/kg) administered intraperitoneally and then killed via heart excision. Hearts were quickly cannulated and placed in a constant-pressure Langendorff tissue bath system (perfusion through the aorta maintained between 60 and 80 mmHg) with warmed (37 °C) and oxygenated (95% O_2_/5% CO_2_) Tyrode’s solution (in mM: 128.2 NaCl, 4.7 KCl, 1.05 MgCl_2_, 1.3 CaCl_2_, 1.19 NaH_2_PO_4_, 20 NaHCO_3_, and 11.1 glucose). The tissue bath housing the heart was customized with an encircled antennae design to provide communication for wireless power transfer and operation of pacing electrodes (Supplementary Fig. 7). Two ECG-sensing electrodes and one ground electrode were placed in the bath around the heart to record far-field ECG signals with LabChart (AD Instruments) throughout experiments. Motion was arrested with an electromechanical uncoupler (10 µM Blebbistatin) and the membrane potential fluorescent dye, di-4-ANEPPS (125 nM) was loaded to optically map voltage changes (ex/em spectra 520/650 nm) in the heart using a complementary metal oxide semiconductor (CMOS) camera with high spatial and temporal resolution (MICAM Ultima, SciMedia), Optical recordings were captured at 2 kHz. Additional information is provided in Supplementary Fig. 8. To wirelessly induce an electrical stimulus onto the isolated mouse heart, the electrical pacing component of the device was placed on the epicardial surface of the right ventricle and the NeuroLux GUI was configured to output pacing stimuli at 10 Hz with 5 ms pulse width using 8 W of power. Capture of electrical stimuli was verified with pseudo ECG recordings and the spatial and temporal spread of electrical activity across the surface of the heart’s surface was recorded with optical mapping.

### Optogenetic pacing

For optostimulation of ex vivo mouse hearts, the same procedure described above was utilized for isolating and perfusing isolated hearts in a temperature-controlled Langendorff system. Optically active ChR2-expressing mouse hearts were specifically used for these studies due to their light-activated cation channels capable of inducing depolarization and action potentials. However, instead of using an electrically charged device, an optically charged device with two µ-ILEDs was placed on the anterior epicardial surface of the right ventricle. To wirelessly induce an optogenetic response in ChR2-expressing mouse hearts, a NeuroLux GUI specifically designed for optical pacing was applied. Specifically, hearts were paced using a pulse duration of 250 ms and frequencies of 5, 6.25, 8.75, and 10 Hz. Frequencies were selected to be faster than the intrinsic heart rate of the Langendorff-perfused mouse heart. Due to the fact that the intermittent blue light pacing interfered with the optical mapping recordings via to spectral overlap, pseudo ECG recordings were predominately used to verify pacing capture in these studies.

### Implantation procedure

All procedures were performed according to the approved protocols by the Institutional Animal Care and Use Committee. Adult rats older than 6 weeks were chosen for the implantation. All procedures were performed under general anesthesia using isoflurane. Animals were intubated using a 16-gauge catheter and ventilated using VentElite Small Animal Ventilator (Harvard Apparatus, Holliston, MA). The heart was exposed via left thoracotomy and the pericardium was sharply excised. For single-electrode devices, the pacemaker electrode was secured on the anterior epicardial surface of the left ventricle (Fig. 3c) using monofilament sutures. For biventricular devices with two electrodes, each electrode leg was sutured to the anterior epicardial surface of the right or left ventricle. The receiver was placed in subcutaneous tissue on the dorsal or ventral aspect of the animal (Fig. 3a, b shows the receiver in the dorsal aspect of the animal). The thoracic cavity, muscle, and skin were closed in subsequent layers. Pacemaker functionality was tested immediately both upon placement of electrodes and following closure. Appropriate postoperative care was provided along with analgesia minimum of 3 days following surgery. Animals were weighed every 3 days for up to 3 weeks (*n* = 3) or 6 weeks (*n* = 3) following implantation.

### Chronic in vivo electrical pacing

In vivo pacing was performed starting at Day 0 of implantation. Each animal was placed inside a circular antenna. The receiver was parallel to the enclosed antenna area, the pacemaker was activated using the NeuroLux software. In our cohorts, the resting heart rate ranged from 300 to 350 b.p.m. The animal underwent ventricular pacing from 400 to 600 b.p.m. during our testing. Ventricular pacing of each animal was evaluated each day in the postoperative period until the device failed to capture the heart. Pacing of awake animals in a freely moving state was performed in the same manner. Additional information is provided in Supplementary Fig. 9.

### Histology

At 0, 3, and 6 week endpoints, rat hearts with implanted devices were excised and perfusion-fixed with 10% neutral buffered formalin fixative. Tissue was paraffin-embedded, sectioned, and stained with Masson’s trichrome. Images were collected using an EVOS XL light microscope (Thermo Fisher Scientific) and the volume fraction of myocytes, collagen, and interstitial space for the cross-sectional area of the anterior left ventricle was determined with a custom MATLAB program. The Kruskal–Wallis test was used to compare the outcome between groups and *p*-values of < 0.05 were taken as statistically significant.

### Mechanics simulation

FEA was used in the commercial software ABAQUS (ABAQUS Analysis User’s Manual 2016). The 75 μm thick PI layer was modeled with hexahedron elements (C3D8R), whereas the 18 μm thick copper and 14 μm thick parylene were modeled with composite shell elements (S4R). The number of elements in the model was ~5 × 106 and the minimal mesh size was ~1/5 of the width of the copper wire (50 µm). The mesh convergence of the simulation was guaranteed for all cases. The elastic modulus (E) and Poisson’s ratio (ν) are EPI = 2.5 GPa, νPI = 0.34; ECu = 119 GPa, νCu = 0.32; EParylene = 2.1 GPa, νParylene = 0.34.

### Thermal finite-element analysis model

ANSYS 19.2 (Software License Agreement Web Version 18 March 2019) was utilized for static-thermal and transient-thermal finite-element modeling, to study the changes in temperature of the epicardial tissue that is in direct contact with the encapsulation surrounding the μ-ILED. Components of the pacemaker, including the copper, PI, and encapsulation layers, were simulated in accurate layouts and using their exact topologies within an octagonal area extending at least 0.95 mm in each direction from the μ-ILED. The model was simulated using Program Controlled Mechanical elements, the minimum edge length of an element being 1/500th (0.1  μm) to ensure mesh convergence. The thermal conductivity, heat capacity, and mass density of the materials used in the simulations was 130 W m^−1^ K^−1^, 490 J kg^−1^ K^−1^, and 8920 kg m^−3^ for the μ-ILED; 0.57 W m^−1^ K^−1^, 4068 J kg^−1^ K^−1^, and 1017 kg m^−3^ for the interstitial fluid; 400 W m^−1^ K^−1^, 385 J kg^−1^ K^−1^, and 8933 kg m^−3^ for the copper traces; 0.56 W m^−1^ K^−1^, 3986 J kg^−1^ K^−1^, and 1081 kg m^−3^ for the epicardial tissue; 0.126 W m^−1^ K^−1^, 837 J kg^−1^ K^−1^, and 1110 kg m^−3^ for the parylene encapsulation; and 0.12 W m^−1^ K^−1^, 1090 J kg^−1^ K^−1^, and 1420 kg m^−3^ for the PI.

### Reporting summary

Further information on research design is available in the Nature Research Reporting Summary linked to this article.

## Supplementary information

Supplementary Information

Description of Additional Supplementary Files

Supplementary Movie 1

Supplementary Movie 2

Supplementary Movie 3

Supplementary Movie 4

Supplementary Movie 5

Supplementary Movie 6

Reporting Summary

## Data Availability

The code for operating the pacemaker of this study are available from the corresponding author upon reasonable request.

## Data availability

Optical mapping data software is publicly available at https://github.com/optocardiography. All raw and processed data will be available upon request.

## References

[1] Klocke R, Tian W, Kuhlmann MT, Nikol S (2007). Surgical animal models of heart failure related to coronary heart disease. Cardiovasc. Res..

[2] Monnet E, Chachques JC (2005). Animal models of heart failure: what is new?. Ann. Thorac. Surg..

[3] Nishida K, Michael G, Dobrev D, Nattel S (2010). Animal models for atrial fibrillation: clinical insights and scientific opportunities. Europace.

[4] Sweeney MO (2003). Adverse effect of ventricular pacing on heart failure and atrial fibrillation among patients with normal baseline QRS duration in a clinical trial of pacemaker therapy for sinus node dysfunction. Circulation.

[5] Wijffels MCEF, Kirchhof CJHJ, Dorland R, Allessie MA (1995). Atrial fibrillation begets atrial fibrillation. Circ. J..

[6] Morillo CA, Klein GJ, Jones DL, Guiraudon CM (1995). Chronic rapid atrial pacing: structural, functional, and electrophysiological characteristics of a new model of sustained atrial fibrillation. Circulation.

[7] Travill CM (1992). Haemodynamic and neurohumoral response in heart failure produced by rapid ventricular pacing. Cardiovasc. Res..

[8] Spinale FG (1991). Collagen remodeling and changes in LV function during development and recovery from supraventricular tachycardia. Am. J. Physiol..

[9] Nussinovitch U, Gepstein L (2015). Optogenetics for in vivo cardiac pacing and resynchronization therapies. Nat. Biotechnol..

[10] Bruegmann T (2016). Optogenetic defibrillation terminates ventricular arrhythmia in mouse hearts and human simulations. J. Clin. Invest..

[11] Nussinovitch U, Shinnawi R, Gepstein L (2014). Modulation of cardiac tissue electrophysiological properties with light-sensitive proteins. Cardiovasc. Res..

[12] Nyns ECA (2017). Optogenetic termination of ventricular arrhythmias in the whole heart: towards biological cardiac rhythm management. Eur. Heart J..

[13] Bruegmann T (2010). Optogenetic control of heart muscle in vitro and in vivo. Nat. Methods.

[14] Wang Y (2017). Optogenetic control of heart rhythm by selective stimulation of cardiomyocytes derived from PNMT+ cells in murine heart. Sci. Rep..

[15] Gutruf P, Good CH, Rogers JA (2018). Perspective: implantable optical systems for neuroscience research in behaving animal models—current approaches and future directions. APL Photonics.

[16] Laughner JI (2013). A fully implantable pacemaker for the mouse: from battery to wireless power. PLoS ONE.

[17] Mickle AD (2019). A wireless closed-loop system for optogenetic peripheral neuromodulation. Nature.

[18] Samineni VK (2017). Fully implantable, battery-free wireless optoelectronic devices for spinal optogenetics. Pain.

[19] Gutruf P (2018). Fully implantable optoelectronic systems for battery-free, multimodal operation in neuroscience research. Nat. Electronics.

[20] Shin G (2017). Flexible near-field wireless optoelectronics as subdermal implants for broad applications in optogenetics. Neuron.

[21] Espe EKS (2013). Novel insight into the detailed myocardial motion and deformation of the rodent heart using high-resolution phase contrast cardiovascular magnetic resonance. J. Cardiovasc. Magn. Reson..

[22] Gutruf P, Walia S, Nur Ali M, Sriram S, Bhaskaran M (2014). Strain response of stretchable micro-electrodes: controlling sensitivity with serpentine designs and encapsulation. Appl. Phys. Lett..

[23] Furrer M (1997). VATS-guided epicardial pacemaker implantation: Hand-sutured fixation of atrioventricular leads in an experimental setting. Surg. Endosc..

[24] Mond H (1988). The porous titanium steroid eluting electrode: a double blind study assessing the stimulation threshold effects of steroid. Pacing Clin. Electrophysiol..

[25] Qian L, Song X, Ren H, Gong J, Cheng S (2004). Mitochondrial mechanism of heat stress-induced injury in rat cardiomyocyte. Cell Stress Chaperones.

[26] Nyns ECA (2019). An automated hybrid bioelectronic system for autogenous restoration of sinus rhythm in atrial fibrillation. Sci. Transl. Med..

[27] Crossley GH (1995). Steroid elution improves the stimulation threshold in an active-fixation atrial permanent pacing lead. A randomized, controlled study. Model 4068 Investigators. Circulation.

[28] Armstrong PW, Stopps TP, Ford SE, de Bold AJ (1986). Rapid ventricular pacing in the dog: pathophysiologic studies of heart failure. Circulation.

[29] deAlmeida, A. C., van Oort, R. J. & Wehrens, X. H. T. Transverse aortic constriction in mice. *J. Vis. Exp.* **38**, e1729 (2010). 10.3791/1729 PMC3164086 20410870

[30] Yutzey KE, Robbins J (2007). Principles of genetic murine models for cardiac disease. Circulation.

[31] Spinale FG (1992). Relation between ventricular and myocyte function with tachycardia-induced cardiomyopathy. Circ. Res..

[32] Spinale FG (1994). Cellular and molecular alterations in the beta adrenergic system with cardiomyopathy induced by tachycardia. Cardiovasc. Res..

[33] Wilson JR (1987). Experimental congestive heart failure produced by rapid ventricular pacing in the dog: cardiac effects. Circulation.

[34] Ravens U (1996). Tachycardia-induced failure alters contractile properties of canine ventricular myocytes. Cardiovasc. Res..

[35] Rademaker MT (1997). Beneficial hemodynamic and renal effects of adrenomedullin in an ovine model of heart failure. Circulation.

[36] Rademaker MT (1996). Natriuretic peptide responses to acute and chronic ventricular pacing in sheep. Am. J. Physiol..

[37] Crocini C (2016). Optogenetics design of mechanistically-based stimulation patterns for cardiac defibrillation. Sci. Rep..

[38] Magnusson P, Liv P (2018). Living with a pacemaker: patient-reported outcome of a pacemaker system. BMC Cardiovasc. Disord..

[39] Gist KM (2019). Cosmetic outcomes and quality of life in children with cardiac implantable electronic devices. Pacing Clin. Electrophysiol..

[40] Bhatia N, El-Chami M (2018). Leadless pacemakers: a contemporary review. J. Geriatr. Cardiol..

