# Supplementary material for: Wireless, battery-free, fully implantable multimodal and multisite pacemakers for applications in small animal models
Source: Nat Commun. 2019 Dec 17;10:5742. doi: 10.1038/s41467-019-13637-w (PMC6917818; doi:10.1038/s41467-019-13637-w)
Supplement: Supplementary file 1 — Supplementary Information [file 41467_2019_13637_MOESM1_ESM.pdf]

SUPPLEMENTARY INFORMATION

Wireless battery-free fully implantable multimodal and multisite pacemakers for applications in small animal models

by Gutruf et al.

Supplementary Figures

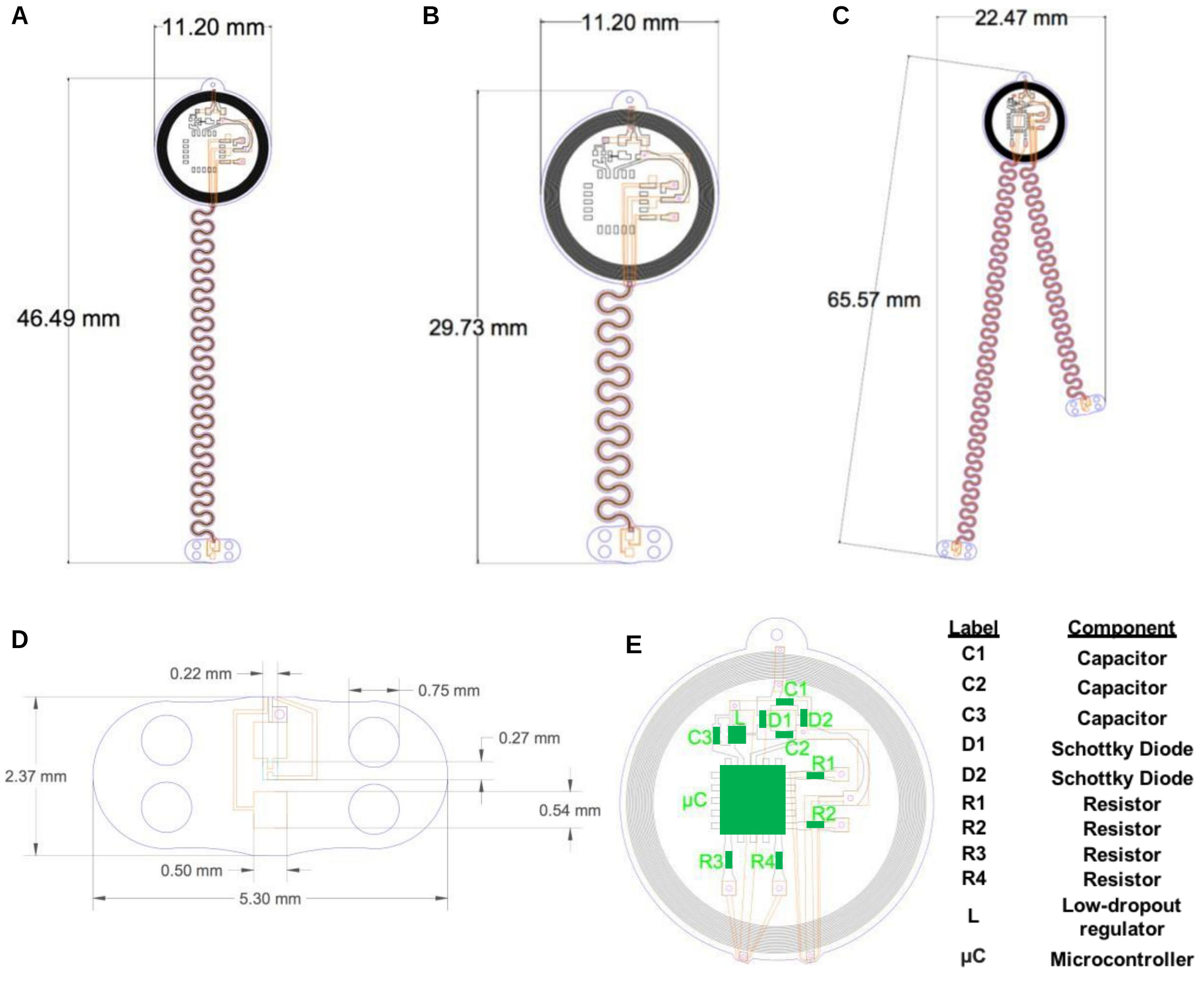

Supplementary Figure 1 Battery-free wireless and fully implantable pacemaker, geometrical and electrical details. (a) Dimensions of single site stimulator device for rats. (b) Dimensions of single site stimulation device for mice. (c) Dimensions of a bilateral device capable of multisite stimulation for rats. (d) Dimensions of the optrode used for electrical and optical stimulation. (e) Electronic components for the bilateral device.

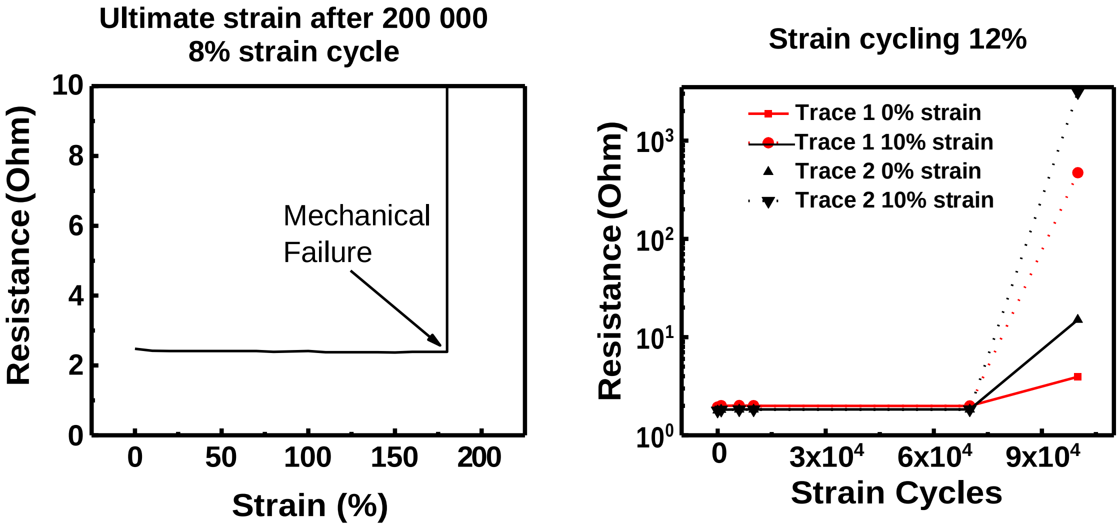

Supplementary Figure 2 **Mechanical failure analysis.** Ultimate electrical and Mechanical strain of electrodes and cycling above elastic limit.

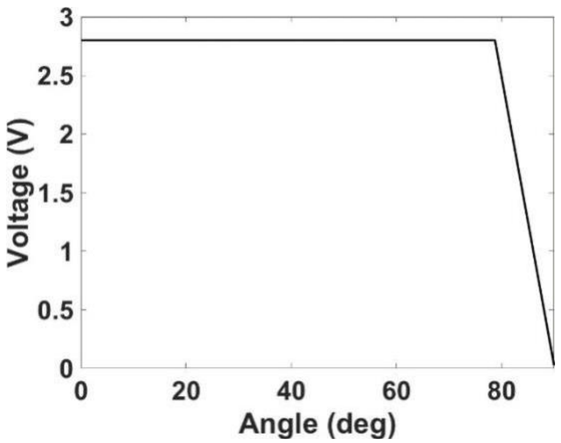

Supplementary Figure 3 **Device power output.** Regulated rectified power at the implant with increasing angle normal to the primary antenna with 3W input power.

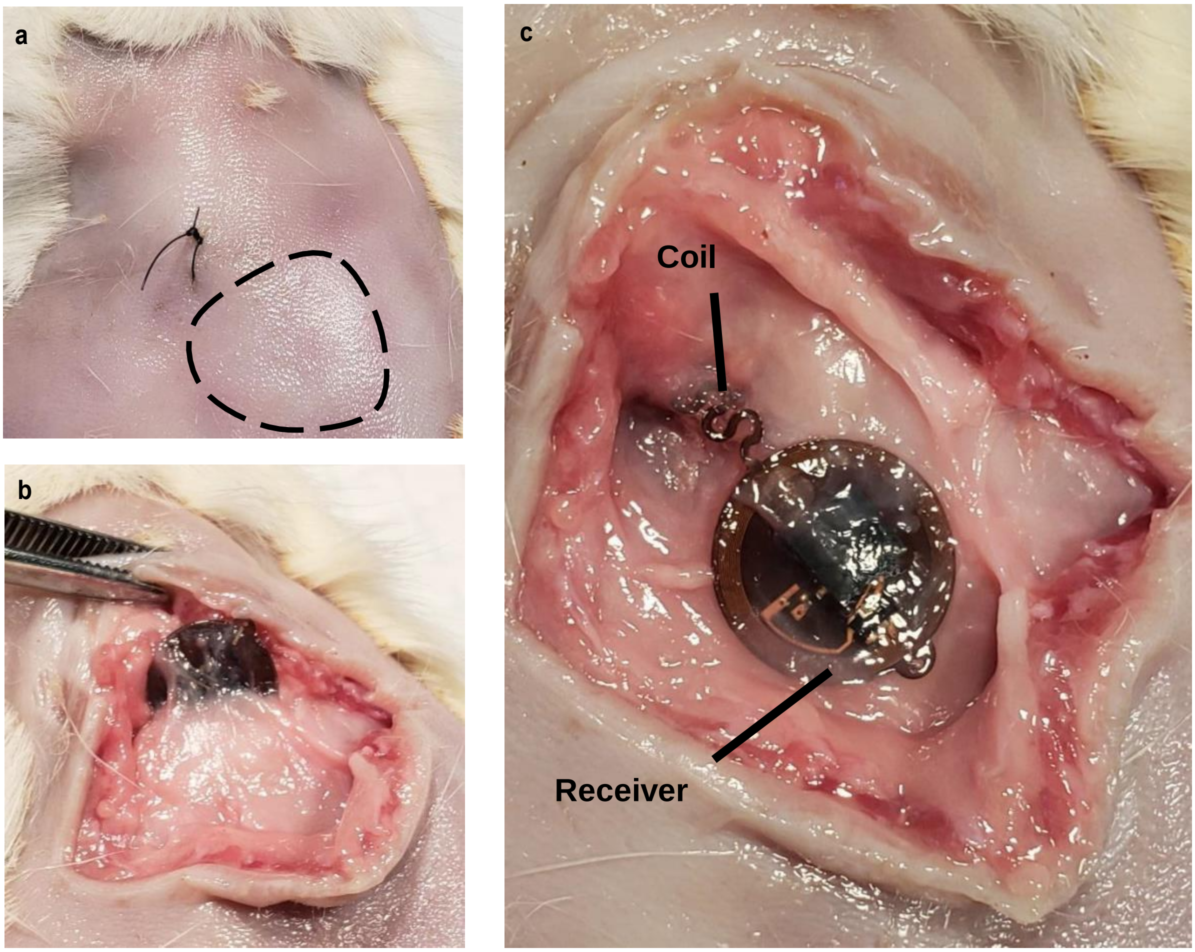

Supplementary Figure 4 Explantation of device at 6 weeks post surgery. The animal is in right lateral decubitus position. (a) Previous incision site is marked by the black suture. The animal has completely healed its incision site. The outline of the receiver is faintly visible which is marked by the dotted line. (b) Device explanation in process. The device is well incorporated into the native tissue. (c) No signs of tissue device rejection or overt inflammatory response is seen during postoperative period. The device and the coil is fully intact at 6 weeks.

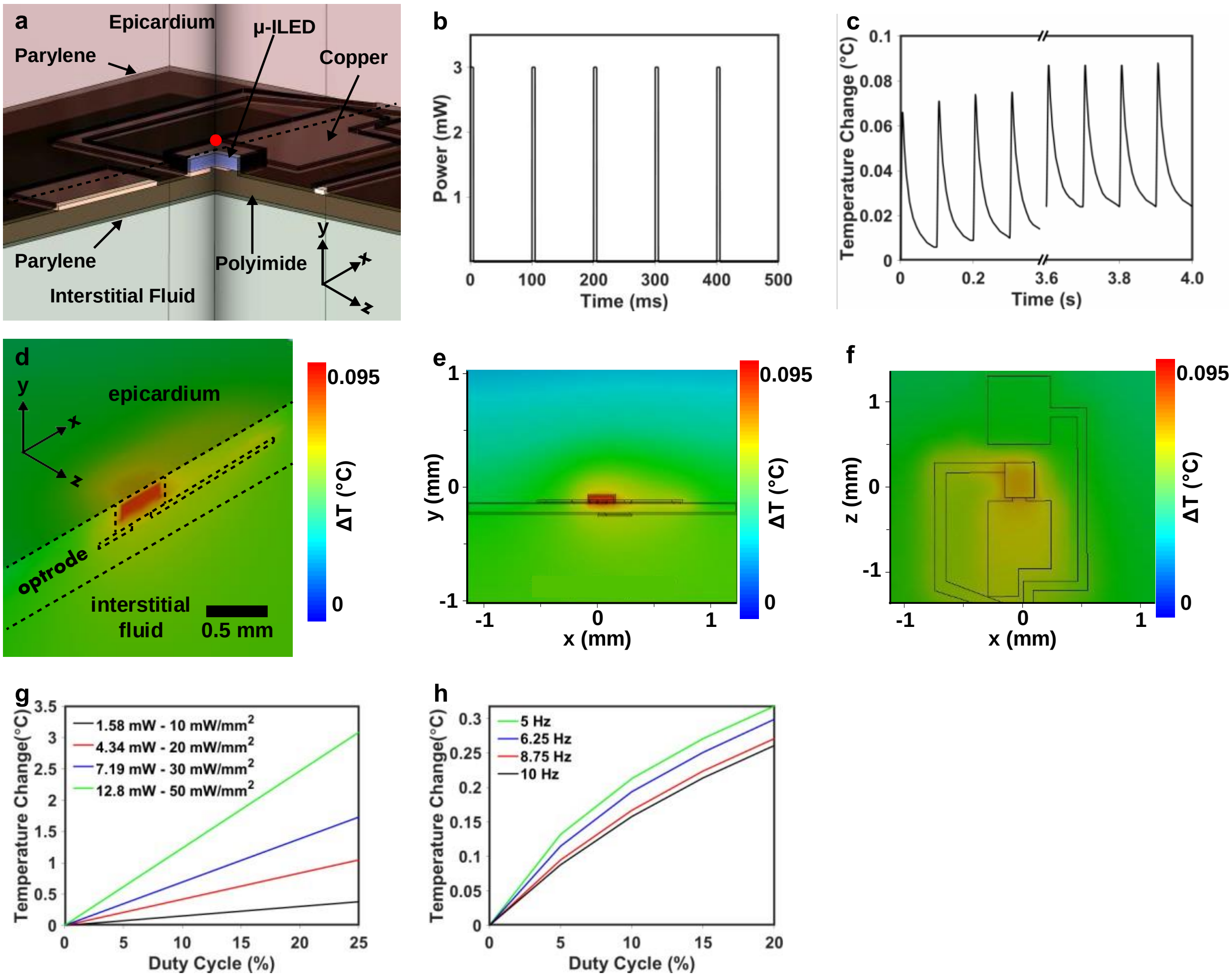

Supplementary Figure 5 Finite element analysis of the heating of cardiac tissue adjacent to a μ- ILED for various operational parameters. (a) 3D cutaway rendering of the model that highlights the detailed layered makeup used for the simulations. The red dot indicates the location of the probe used to record temperature for results presented in frames (c), (g), and (h). (b) Time resolved electrical power for an μ-ILED during operation in a unilateral pacing mode. The peak values are 3 mW electrical power, corresponding to an irradiance of 10 mW/ mm2. (c) Temperature at the interface between the probe and the cardiac tissue as a function of time for operational conditions shown in frame (b). (d) 3D cutaway image of changes in temperature through the mid-line cross section of the optrode, indicated in panel (a), epicardium, and interstitial fluid for steady state operation with thermal input (3 mW). (e), (f) 2D cross sections in X-Y and Z-X

planes respectively with operating conditions analog to (d). (g) Steady state increase in local temperature at the probe location as a function of thermal power at 10 Hz for pulsed operation as illustrated in panel (b). (h) Steady state change in temperature at the site of the probe as a function of increasing temperature and duty cycle with a constant irradiance of 10mW/mm2.

a

b

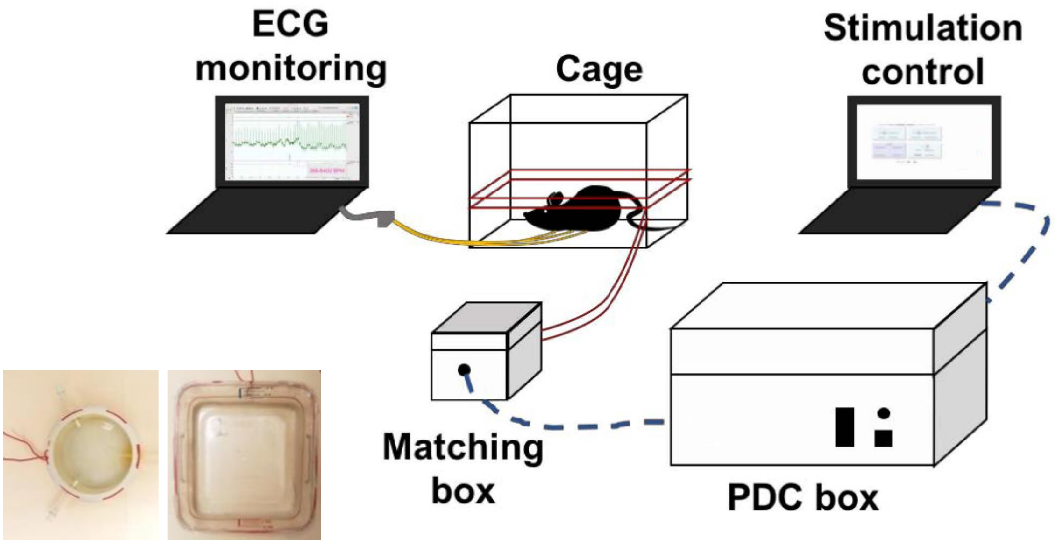

Supplementary Figure 6 **In vivo stimulation system set-up.** (a) Full system set-up. (b) Modular antenna allows for customized power transfer chambers.

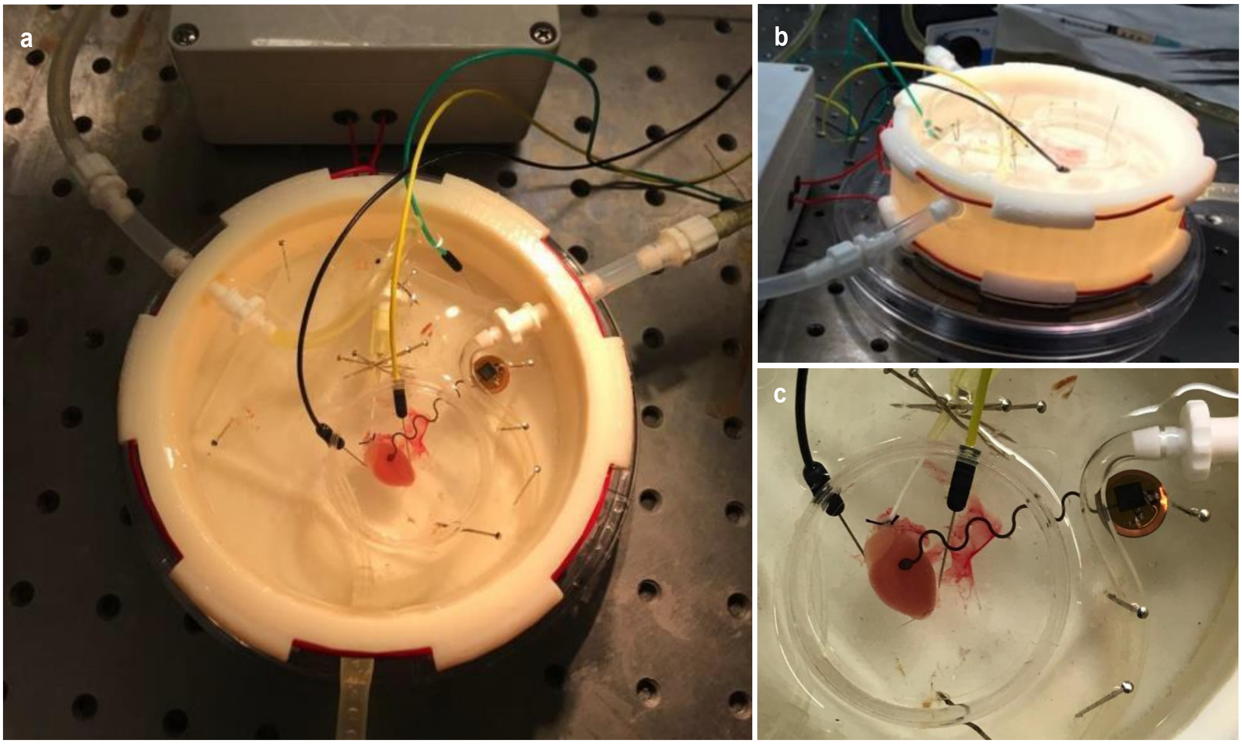

Supplementary Figure 7 Langendorff-perfused heart system set-up. (a) NeuroLux pacing set-up with custom chamber for Langendorff-perfused heart. (b) Side view of customized wireless power transfer chamber with NeuroLux pacing system. (c) Placement of device onto anterior epicardial surface of a mouse heart.

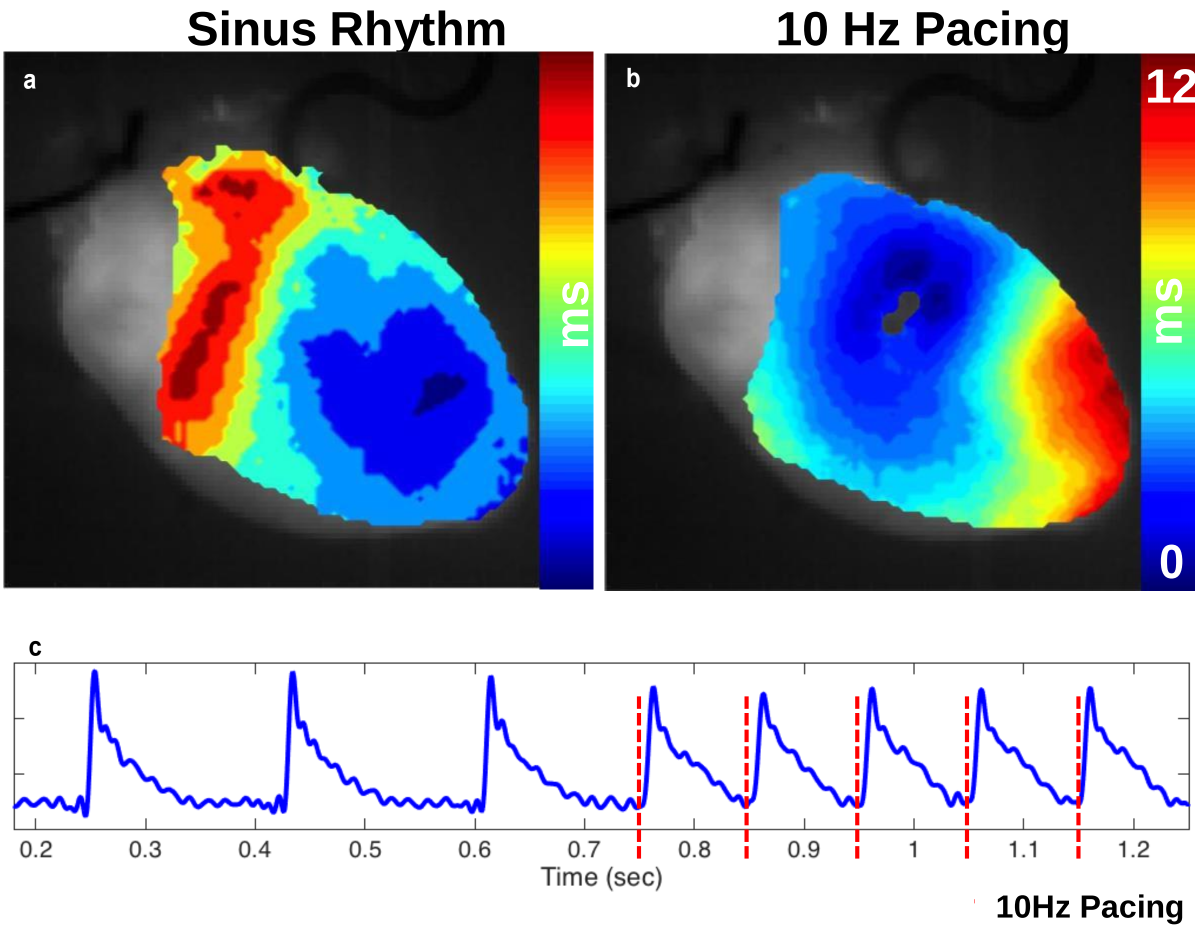

Supplementary Figure 8 Activation for ex vivo electrical pacing. (a) Electrical activation of membrane potential during normal sinus rhythm. (b) Electrical activation of membrane potential during 10 Hz electrical pacing using device. (c) Electrical activation captured for ex vivo pacing at 10 Hz.

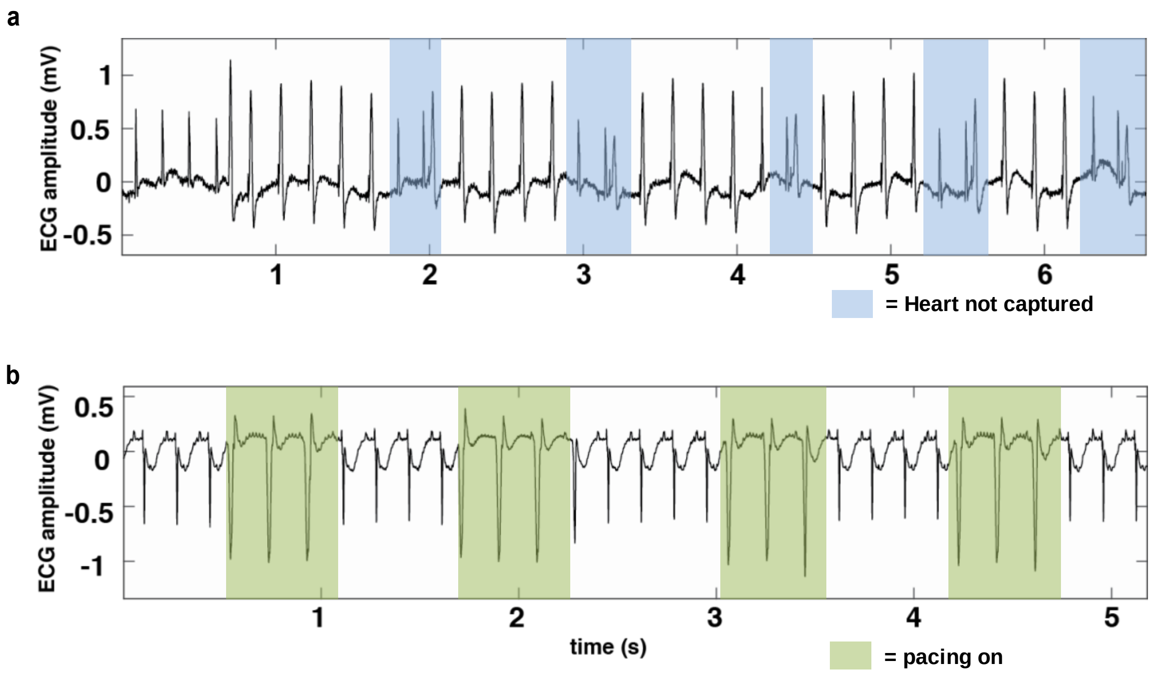

Supplementary Figure 9 Additional in vivo pacing functionality. (a) Inconsistent pacing is not able to fully capture the heart after Day 6 in vivo pacing but device is still able to deliver electrical stimuli. (b) On/off settings of pacemaker is easily toggled in the order of seconds.
