# Supplementary material for: Wireless, battery-free, fully implantable multimodal and multisite pacemakers for applications in small animal models
Source: Nat Commun. 2019 Dec 17;10:5742. doi: 10.1038/s41467-019-13637-w (PMC6917818; doi:10.1038/s41467-019-13637-w)
Supplement: Supplementary file 2 — Description of Additional Supplementary Files [file 41467_2019_13637_MOESM2_ESM.pdf]

Description of Additional Supplementary Files

File Name: Supplementary Movie 1
Description: **Ex vivo electrical pacing.** Ex vivo electrical pacing of a mouse heart shows a visual increase in the rate of mechanical contractions.

File Name: Supplementary Movie 2
Description: **Ex vivo electrical pacing activation map.** Activation map of ex vivo electrical pacing demonstrates anisotropic propagation of the membrane potential originating from the middle of the right ventricle.

File Name: Supplementary Movie 3
Description: **In vivo live pacing.** In vivo live pacing of a rat heart

File Name: Supplementary Movie 4
Description: **Ex vivo optical pacing.** Ex vivo optical pacing of a ChR2-expressing mouse heart

File Name: Supplementary Movie 5
Description: **Heat transfer simulation – bottom view.** Time resolved video of heat transfer simulation bottom view for optical pacing of the optrode at 10 Hz and optical output of 10mW/mm2.

File Name: Supplementary Movie 6
Description: **Heat transfer simulation – side view.** Time resolved video of heat transfer simulation side view for optical pacing of the optrode at 10 Hz and optical output of 10mW/mm2.
