# Supplementary material for: Wireless, battery-free, fully implantable multimodal and multisite pacemakers for applications in small animal models
Source: Nat Commun. 2019 Dec 17;10:5742. doi: 10.1038/s41467-019-13637-w (PMC6917818; doi:10.1038/s41467-019-13637-w)
Supplement: Supplementary file 9 — Reporting Summary [file 41467_2019_13637_MOESM9_ESM.pdf]

nature research

Corresponding author(s): Philipp Gutruf, Igor Efimov, John A Rogers

Last updated by author(s): Oct 28, 2019

Reporting Summary

Nature Research wishes to improve the reproducibility of the work that we publish. This form provides structure for consistency and transparency in reporting. For further information on Nature Research policies, see Authors & Referees and the Editorial Policy Checklist .

Statistics

For all statistical analyses, confirm that the following items are present in the figure legend, table legend, main text, or Methods section.

n/a	Confirmed	
☐	☒	The exact sample size (*n*) for each experimental group/condition, given as a discrete number and unit of measurement
☐	☒	A statement on whether measurements were taken from distinct samples or whether the same sample was measured repeatedly
☐	☒	The statistical test(s) used AND whether they are one- or two-sided<br>*Only common tests should be described solely by name; describe more complex techniques in the Methods section.*
☒	☐	A description of all covariates tested
☐	☒	A description of any assumptions or corrections, such as tests of normality and adjustment for multiple comparisons
☐	☒	A full description of the statistical parameters including central tendency (e.g. means) or other basic estimates (e.g. regression coefficient) AND variation (e.g. standard deviation) or associated estimates of uncertainty (e.g. confidence intervals)
☐	☒	For null hypothesis testing, the test statistic (e.g. *F*, *t*, *r*) with confidence intervals, effect sizes, degrees of freedom and *P* value noted<br>*Give P values as exact values whenever suitable.*
☒	☐	For Bayesian analysis, information on the choice of priors and Markov chain Monte Carlo settings
☒	☐	For hierarchical and complex designs, identification of the appropriate level for tests and full reporting of outcomes
☒	☐	Estimates of effect sizes (e.g. Cohen's *d*, Pearson's *r*), indicating how they were calculated

Our web collection on statistics for biologists contains articles on many of the points above.

Software and code

Policy information about availability of computer code

Data collection	ECG data collection was performed using LabChart software (AD Instruments), and optical mapping data was collected using MiCAM Ultima High Spped Imaging System (SciMedia).
Data analysis	A custom MATLAB code, RHYTHM, was used to analyze the optical mapping data on MATLAB R2017A. RHYTHM is available at https://github.com/optocardiography. Raw data of ECG traces were exported from LabChart.

For manuscripts utilizing custom algorithms or software that are central to the research but not yet described in published literature, software must be made available to editors/reviewers. We strongly encourage code deposition in a community repository (e.g. GitHub). See the Nature Research guidelines for submitting code & software for further information.

Data

Policy information about availability of data

All manuscripts must include a data availability statement. This statement should provide the following information, where applicable:

- Accession codes, unique identifiers, or web links for publicly available datasets
- A list of figures that have associated raw data
- A description of any restrictions on data availability

Optical mapping data software is publicly available at https://github.com/optocardiography. All raw and processed data will be available upon request.

Field-specific reporting

Please select the one below that is the best fit for your research. If you are not sure, read the appropriate sections before making your selection.

☒ Life sciences ☐ Behavioural & social sciences ☐ Ecological, evolutionary & environmental sciences

Life sciences study design

All studies must disclose on these points even when the disclosure is negative.

Sample size	For in vivo demonstrations, the sample size was determined by the adequacy of feasible and consistent stimulation across animals (n=13). Ex vivo demonstrations required only 1 successful demonstration because of the proof-of-concept nature (n=1). For histological analysis, 3 animals were assessed at each of the three time end points in order to have an adequate sample size while also optimizing use of animals (n=9).
Data exclusions	Data from animals used in device design optimization were excluded.
Replication	Stimulation was replicated multiple times in all studied animals at different time points and various stimulation frequencies. Ex vivo stimulation was replicated using the same optical mapping protocol.
Randomization	Randomization was not required in the study.
Blinding	Personnel completing quantitative analysis of the Masson's trichrome stain were blinded to experimental groups. For optical mapping experiments, blinding was not possible because the experimental results are evident from the image data.

Reporting for specific materials, systems and methods

We require information from authors about some types of materials, experimental systems and methods used in many studies. Here, indicate whether each material, system or method listed is relevant to your study. If you are not sure if a list item applies to your research, read the appropriate section before selecting a response.

Materials & experimental systems

n/a	Involved in the study
☒	☐ Antibodies
☒	☐ Eukaryotic cell lines
☒	☐ Palaeontology
☐	☒ Animals and other organisms
☒	☐ Human research participants
☒	☐ Clinical data

Methods

n/a	Involved in the study
☒	☐ ChIP-seq
☒	☐ Flow cytometry
☒	☐ MRI-based neuroimaging

Animals and other organisms

Policy information about studies involving animals; ARRIVE guidelines recommended for reporting animal research

Laboratory animals	Rats, Sprague-Dawley, male and female, adults
Wild animals	The study did not involve wild animals.
Field-collected samples	The study did not involve samples collected from the field.
Ethics oversight	The George Washington University Institutional Animal Care and Use Committee

Note that full information on the approval of the study protocol must also be provided in the manuscript.
